# Supplementary material for: Carotid artery stenosis and ischemic cerebrovascular events after radiotherapy in patients with head and neck cancer
Source: PLoS One. 2025 Jan 30;20(1):e0314861. doi: 10.1371/journal.pone.0314861 (PMC11781643; doi:10.1371/journal.pone.0314861)
Supplement: Attachment — Submitted filename: PONE-D-24-33754 Reviewers Question.pdf [file pone.0314861.s001.pdf]

PONE-D-24-33754

Abstract

1. The abstract mentions relative risks (RR) for various factors associated with carotid artery stenosis (CAS) (e.g., RR of 2.32 for laryngeal cancer and RR of 2.26 for age >65 years), but it does not provide 95% confidence intervals and p values.

Study Design and Methodology

1. The manuscript describes that 907 patients were enrolled. What was the strategy for addressing missing data from patients who could not complete follow-up? How might this impact the reliability of the results?
2. The study has a cross-sectional design, but it follows patients longitudinally through to the end of 2023. Is there a rationale for not utilizing a cohort or case-control design, which may have offered more robust data on causality?
3. The authors note that laboratory data was incomplete, limiting statistical analysis. How significant are these missing data for the study's conclusions, especially in understanding the risk factors? Could imputation methods have been used to address this?

Statistical Analysis

1. The study uses a generalized linear model to calculate risk ratios, but the rationale for this choice is not discussed. Why was this model chosen over alternative models such as Cox proportional hazards, especially given that survival analysis is also performed? Can the authors provide further justification or compare the results with alternative modeling techniques?
2. Although some potential confounders, such as age and comorbidities, are addressed, the manuscript does not clearly indicate how interactions between variables were managed. Were interactions between key risk factors like age, cancer type, and diabetes mellitus evaluated?
3. The follow-up duration varies, and events such as carotid artery stenosis (CAS) and ischemic cerebrovascular events (ICVE) are time-dependent. Would the results be affected if sensitivity analyses were performed to control for different follow-up times across patients? Was a time-to-event analysis considered?

Results and Discussion

1. The manuscript references several studies showing a higher incidence of CAS and ICVE post-radiotherapy in head and neck cancer patients. How do the authors reconcile the discrepancy between their lower reported rates and those in previous studies? Could differences in radiation dosages or imaging modalities used for detection account for this?
2. The study finds a significant increase in wall thickness and plaque scores post-radiotherapy. Is there a biological explanation for these findings beyond what is already known? Could specific biomarkers of radiation-induced vascular injury have been explored to add depth to this analysis?
3. The study focuses on a Thai population. How applicable are the results to other ethnic groups, especially in terms of radiation dosages, healthcare access, and comorbidity profiles? Can the authors discuss this limitation more thoroughly?
4. The manuscript mentions potential mechanisms of radiation-induced vascular damage but could benefit from a deeper discussion of current advancements in radiobiology. For

example, what role might inflammation or endothelial dysfunction play in exacerbating CAS after radiotherapy?

5. The study suggests vascular surveillance, but there is no mention of the cost-effectiveness or practicality of implementing routine surveillance in clinical practice. How feasible would it be to integrate these recommendations into standard care for head and neck cancer patients, particularly in resource-limited settings?
6. The discussion section outlines some limitations, such as incomplete follow-up for some patients and lack of standardization in CT protocols. However, were there any biases introduced by the retrospective nature of the study, especially in clinical decision-making during follow-up care?
